# Measurement of the Inner Retinal Layers of Megalopapilla by Optical Coherence Tomography

**Published:** 2017

**Authors:** Rita GAMA, Catarina RELHA, Joana GOMES COSTA, Nuno EIRO

**Affiliations:** 1 Hospital da Luz, Lisbon 1500, Portugal

**Keywords:** Megalopapilla, Large Cup Disc, Peripapillary Retinal Nerve Fiber Layer Thickness, Ganglion Cell Inner Plexiform Layer Thickness, Cirrus Spectral Domain Optical Coherence Tomography, Distance Optic Nerve Border, Glaucoma, Lamina Cribrosa, Interpore connective tissue, Preperimetrical Glaucomatous Lesion

## Abstract

The main purpose of this study was to assess the differences in the peripapillary retinal nerve fiber layer (pRNFL) and ganglion cell-inner plexiform layer (GCIPL) thicknesses between subjects with megalopapilla (MP) and those with large (physiological) cup discs (LCD) measured by spectral-domain optical coherence tomography. The secondary purpose was to determine whether pRNFL and GCIPL thicknesses increase with the optic nerve head (ONH) area. This cross-sectional study included 184 eyes (92 eyes with MP and 92 eyes with LCD). The subjects with LCD were used as sex-and-age-matched controls. All subjects were imaged using the Cirrus HD-OCT system. Macula and pRNFL thickness maps were obtained for all subjects. The inferior quadrant pRNFL thickness was higher in the MP group than in the LCD group (P < 0.05). There were no differences in the GCIPL thickness between the two groups. A positive correlation was found between average, superior, and inferior quadrant pRNFL thicknesses and the ONH area (P < 0.05). The slope of the correlation curve was higher for the inferior quadrant. No correlation was found between the GCIPL thickness and the ONH area. In comparison to patients with LCD, the inferior quadrant pRNFL thickness of patients with MP was higher. As the ONH area increased, the average, superior, and inferior quadrant pRNFL thicknesses also increased. In patients with MP, the assessment of a glaucomatous lesion based on pRNFL thickness measurements may not be reliable. It is recommended that in these patients, the evaluation of glaucomatous damage be based on the GCIPL thickness map analysis rather than on the pRNFL thickness.

## INTRODUCTION

**Figure 1:**
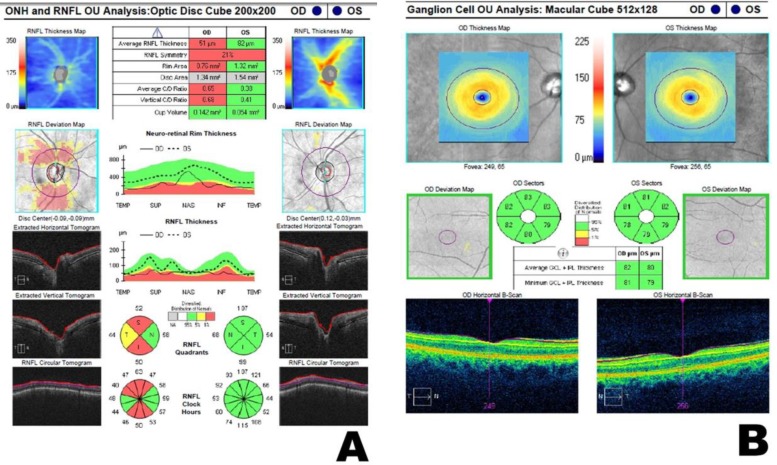
Cirrus HD-OCT Analysis of the Peripapillary Retinal Nerve Fiber Layer (pRNFL) Thickness (A) and Ganglion Cell-Inner Plexiform Layer (GCIPL) Thickness (B). In A, the Table at the Top Shows the Stereometric Optic Nerve Parameters and the Graphs at the Bottom show the Quadrant and Clock Hour Distribution of the pRNFL Thickness. All the Parameters were measured around the Optic Nerve Head. In B, the Table shows the Average and Minimum GCIPL Thicknesses and the Circle Graphs show the Distribution of Sectorial Thicknesses. All these Measurements were taken at the Macula. The Graphs are colored according to the Normal Distribution for Age and Sex.

The innermost retinal layers one can identify on optical coherence tomography (OCT) imaging are the retinal nerve fiber layer (RNFL), which corresponds to the axons of the ganglion cell and, immediately outwards, the ganglion cell-inner plexiform layer (GCIPL) composed of ganglion cell bodies [1]. Although they represent parts of the same cell, the measurement of their thicknesses is taken at different locations by OCT. The GCIPL thickness is measured at the macula, where the concentration of cell bodies is higher [2]. In turn, the RNFL thickness is measured at the center of the optic disc, which is the reason why it is designated peripapillary RNFL or pRNFL (Fig 1).

**Figure 2:**
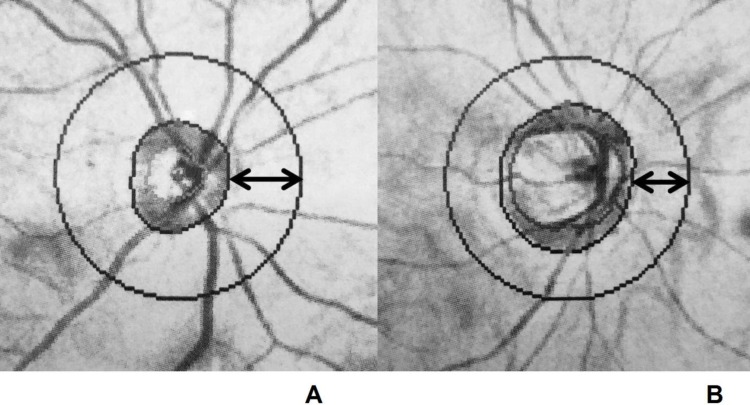
The Estimated Distance (arrow) of the Scan to the Optic Nerve Head Border during Peripapillary Retinal Nerve Fiber Layer Thickness Measurements. The Diameter of the Scan is 3.46 mm. A - On a Normal Disc with an Area of 1.83 mm^2^, the Distance is 967 μm. B - On a Large Disc with an Area of 3.19 mm^2^ (Megalopapilla), the Distance is 722 m.d = 1.73 − $\sqrt[{2}]{a/\pi }$ (Calculated by the Authors Based on the Principle that the Area Enclosed by a Circle (a) of radius (r) is π r^2^)

The Cirrus HD-OCT system provides measurements of the pRNFL and GCIPL thicknesses that represent a crucial step in differentiating between normal and glaucomatous eyes. Patients with megalopapilla (MP; optic nerve head [ONH] area larger than 2.50 mm^2^) have a thicker pRNFL [3-8]. It is not clear if this increased thickness represents a higher number of retinal nerve fibers or is an error of measurement attributed to the fixed diameter of the scan (3.46 mm). Some authors believe that, in subjects with large (physiological) cup discs (LCD), there is an overestimation of the pRNFL thickness, since the measurements by OCT are taken closer to the ONH edge (Fig 2) [3, 8].

If this overestimation is proved to be real, the measurements of pRNFL thickness in MP patients with glaucoma may not be reliable. The GCIPL thickness in subjects with LCD should also be considered because measurements at the macula are not influenced by the diameter of the scan. The main purpose of this study was to compare pRNFL and GCIPL thicknesses measured by Cirrus HD-OCT between subjects with MP and those with LCD. The secondary purpose was to determine whether the pRNFL and GCIPL thicknesses increase with the ONH area.

a = Optic Nerve Head Area (mm^2^)

d = Distance of the Scan to the Optic Nerve Head Border

The Photographs were taken by the Authors.

## MATERIALS AND METHODS

This cross-sectional study included subjects attending our department of ophthalmology between February 2013 and July 2015. Ethics committee approval was obtained and the study protocol followed the statements of the Declaration of Helsinki. Written informed consent was obtained from all subjects before their inclusion in the study. The study subjects were divided into two groups: those with MP, with an ONH area >2.5 mm^2^; and those with LCD, with ONH area <2.4 mm^2 ^and average disc/cup ratio <0.55. The subjects with LCD were used as sex-and-age-matched controls. MP can be a unilateral or bilateral finding. In the case of unilateral MP, an LCD control was found, and subjects were matched according to sex and age (with a difference of 11 months or less). For bilateral MP, the right eye was included in the study and an LCD control was found as per the same matching criteria. All subjects underwent a complete ophthalmic examination (including biomicroscopy, intraocular pressure measurement, and fundus examination). If any abnormality was found, the subject was excluded from the study. Standard automated perimetry using the Swedish Interactive Threshold algorithm and the 30-2 program (Humphrey Field Analyzer; Carl Zeiss Meditec Inc., Dublin, CA) was performed in all participants. Subjects were included if visual fields were normal and reliable (fixation losses and false-positive and false-negative responses <33%). Good images on Cirrus HD-OCT were defined by signal strength ≥7/10 and patients were included if they agreed to participate in the study. Subjects were excluded if there was a refractive error exceeding 2.0 D sphere and/or 1.5 D cylinder on autorefractometer measurements, any media opacity preventing imaging techniques, abnormalities of the disc or macular disorders, or an inability to undergo the test. Cirrus HD-OCT imaging was performed by the authors on all subjects.

pRNFL Thickness

The pRNFL thickness was obtained by an optic disc cube 200 × 200 scan. Correct centration and pRNFL segmentation were checked for each OCT image. Images that were obtained during visible eye motion or blinking artifacts, that were unfocused, and those that were poorly centered were excluded. After manual centration at the disc, the Cirrus HD-OCT system calculates the pRNFL thickness on a specific location defined by a circle with 3.46 mm in diameter. The average pRNFL thickness, pRNFL thickness of each quadrant (temporal, superior, nasal, and inferior), and individual pRNFL thickness of all clock hour sectors are calculated based on the measurements that are taken in this circle [9-11]. Clock hour, quadrant, and total average RNFL thicknesses are represented in colored graphs where green represents values within 95% normal distribution, yellow, within the lower 1%–5% of normal distribution, and red, within the lower 0%–1% of normal distribution, with reference to a normative database (Fig 1A) [9-11]. Exams were excluded if there was a defect at the 5% level (in yellow or red) on the pRNFL thickness deviation quadrants map, more than 1 hour defect in the pRNFL thickness deviation clock hours map, or a poor quality image.

GCIPL Thickness

The ganglion cell analysis was measured in the macular cube 512 × 128 scan mode for detection of retinal disorders. The center of the scan circle was automatically located at the fovea in each case. For this analysis, the algorithm identifies the GCIPL (from the outer border of the pRNFL to the outer border of the inner plexiform layer [IPL] including the retinal ganglion cell [GC] layer and the IPL). The average, minimum (lowest GCIPL thickness measured), and sectorial (superotemporal, superior, superonasal, inferonasal, inferior, and inferotemporal) thicknesses of the GCIPL were measured in an elliptical annulus (an area defined by two ellipses, the outer ellipse with vertical and horizontal radius of 2.0 and 2.4 mm, respectively; and the inner ellipse with vertical and horizontal radius of 0.5 and 0.6 mm, respectively). Sector, minimum, and total average GCIPL thicknesses were coded in color graphs with green, yellow, and red, similar to the pRNFL (Fig 1B) [11, 12].

In this study, data were analyzed using the statistical package SPSS for Windows (version 18.0; SPSS Inc., Chicago, IL, USA). The continuous variables were compared among groups using an independent-samples t-test. The chi-square and Kruskal–Wallis tests were used to compare discrete variables between two groups. Correlations between the pRNFL and GCIPL thicknesses and the ONH area were analyzed using the ANOVA correlation for nonparametric data. The bias was evaluated statistically as the mean of the differences compared with zero. The 95% limits of agreement were also calculated and p-values of less than 0.05 were considered statistically significant. 

**Table 1:** Measurement of Peripapillary Retinal Nerve Fiber Layer (pRNFL) Thickness and Ganglion Cell-Inner Plexiform Layer (GCIPL) Thickness by Cirrus HD-OCT in Megalopapilla (MP) and large (physiological) Cup Disc (LCD) Groups

	LCD group (mean SD)	MP group (mean SD)	P
Age (years)	57.64 14.18 (20–85)	57.70 14.15 (21–86)	0.956
Females, n (%)	65 (70.7%)	64 (69.6%)	-
pRNFL quadrant thickness (m)			
** Superior**	112.93 16.23	115.85 14.94	0.207
** Nasal **	74.15 10.73	73.95 12.07	0.902
** Inferior **	117.35 18.69	124.10 17.85	0.013
** Temporal **	66.60 11.67	64.04 10.02	0.113
** Average **	92.59 10.43	94.74 9.88	0.152
GCIPL thickness (m)			
** Average **	79.43 7.18	79.40 7.00	0.975
** Minimum **	76.55 8.11	76.24 9.44	0.808
** Superior sector**	79.18 8.15	79.36 8.71	0.889
** Superonasal sector **	81.11 7.84	80.27 7.83	0.470
** Inferonasal sector **	79.77 7.99	79.39 6.93	0.740
** Inferior sector **	77.83 8.29	78.25 6.93	0.707
** Inferotemporal sector **	79.87 7.48	80.45 6.72	0.583
** Superotemporal sector **	78.53 7.03	78.33 7.66	0.849

Inferior quadrant pRNFL thickness was significantly different between the two groups.

**Figure 3:**
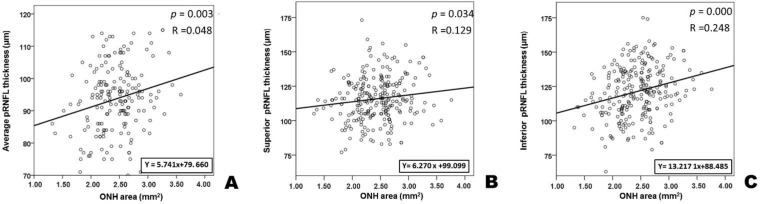
Scatter Plots for the Peripapillary Retinal Nerve Fiber Layer (pRNFL) Thickness and the Optic Head Nerve (OHN) Area

A: Average pRNFL Thickness (y = 5.741x + 79.6060, P = 0.003, R = 0.048); B: Superior Quadrant pRNFL Thickness (y = 6.270x + 99.009, P = 0.04, R = 0.129); and C: Inferior Quadrant pRNFL Thickness (y = 13.217x + 88.485, P = 0.000, R = 0.248).

## RESULTS

This study included 184 eyes (92 eyes with MP and 92 eyes with LCD). Fifty-four subjects had unilateral MP, and 38 had bilateral MP. The mean age of the MP group was 57.70 14.15 years (20–85 years) and that of the LCD group was 57.64 14.18 years (21–86 years); 129 (70.1%) of the eyes belonged to females (Table 1). The age distribution did not significantly differ between the groups (P = 0.956). 

**Figure 4:**
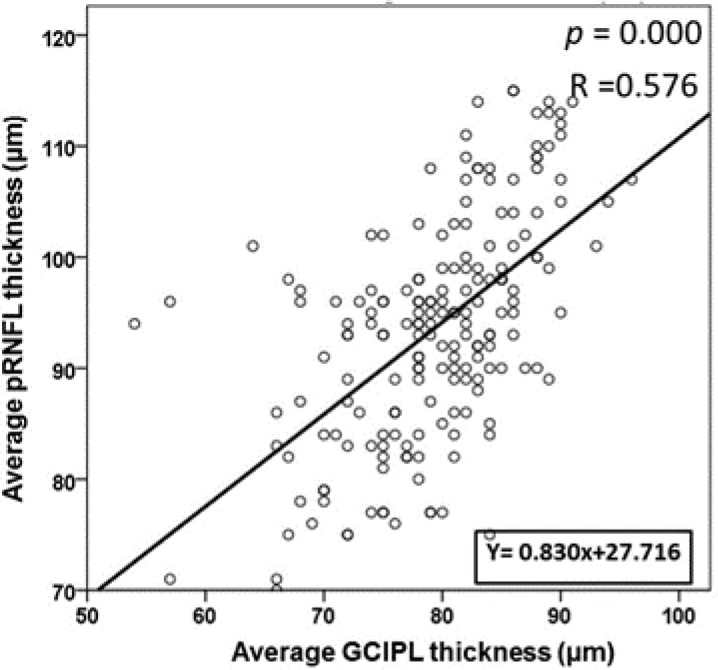
Scatter Plots for the Peripapillary Retinal Nerve Fiber Layer (pRNFL) and Ganglion Cell-Inner Plexiform Layer (GCIPL) Thicknesses (y = 0.830x + 27.716, P = 0.000, R = 0.576)

Inferior quadrant pRNFL thickness was significantly higher in the MP group than in the LCD group (Table 1). No differences on GCIPL thickness were found between the two groups. There was a positive correlation between average, superior, or inferior quadrant pRNFL thickness and the ONH area (P = 0.003 for average pRNFL thickness, P = 0.034 for superior quadrant pRNFL thickness, and P = 0.000 for inferior quadrant pRNFL thickness) (Fig 3).

A positive correlation between pRNFL and GCIPL thicknesses was also found for all subjects (P = 0.000) (Fig 4). No correlation was found between temporal or nasal pRNFL and all GCIPL sector thicknesses with the ONH area.

## DISCUSSION

The main findings of this study were the differences on the inferior quadrant pRNFL thickness between MP and LCD eyes and a positive correlation of superior, inferior, or average pRNFL thickness and the ONH area. To our knowledge, this is the first study that compares pRNFL and GCIPL thicknesses between MP and LCD. In agreement with the findings of this study, Savini and Funaki found that Stratus OCT measurements of average and quadrant pRNFL thicknesses are significantly increased in patients with MP [7, 8]. Savini and Funaki used Time Domain technology and the ONH area was calculated indirectly. Both studies have included a small number of patients with MP. They suggested that an overestimation of pRNFL thickness could happen in patients with MP because the OCT measurements were taken closer to the optic disc edge. The absence of correlation between GCIPL thicknesses with the ONH area was a surprising result. Since pRNFL and GCIPL represent different parts of the same cell, it would be expected that the increased number of retinal fibers within the ONH area would also represent an increased number of ganglion cell bodies (Fig 4). In histological studies, Jonas found a higher proportion of the interpore connective tissue in total lamina cribrosa tissue in larger discs compared with small discs [13]. Jonas also found that the mean single pore area and summed pore area were significantly larger in the superior and inferior regions than in the temporal and nasal quadrants. On histological micrographs, the nerve fiber density at the optic nerve is significantly higher in eyes with microdiscs than in eyes with large ONH [13, 14]. The only plausible explanation for the increased thickness of the pRNFL with the ONH area without a corresponding increased thickness of the GCIPL, is a larger space between the fibers, as the result of a thicker interpore connective tissue, especially in superior and inferior quadrants. In large discs, this organization at the lamina cribrosa could influence the amount of space between the retinal fibers near the optic nerve edge, where the measurements are taken. The effect is more pronounced on the inferior quadrant than on the superior quadrant, where the steepness of the correlation curve is higher (y = 13.217x + 88.485 and y = 6.270x + 99.009, respectively). That should be the reason for a statistical significant difference that was found between MP and LCD only on the inferior quadrant. Preperimetrical glaucomatous lesions are defined on HD-OCT as a decreased thickness of superior and inferior quadrants of the pRNFL. There is a topographic correlation between pRNFL and ganglion cell-inner plexiform layer (GCIPL) thickness defects on OCT [11, 12, 15-19]. We showed that in normal MP, there is an increased pRNFL thickness on the same quadrants where the glaucoma defects are found. These results suggest that in patients with MP, the assessment of a glaucomatous lesion based on the pRNFL thickness analysis may not be reliable. Therefore, we recommend that a careful evaluation of glaucoma damage by Cirrus HD-OCT in patients with MP privileges the GCIPL thickness map analysis rather than the pRNFL thickness. In conclusion, our study shows that using Cirrus HD-OCT, the inferior quadrant pRNFL thickness is higher in MP than in LCD and there is a positive correlation between average, superior, or inferior quadrants of pRNFL thickness and the ONH area. No correlation was found between GCIPL thickness and the ONH area. As discussed above, in patients with MP, the assessment of a glaucomatous lesion based on pRNFL thickness measurements may not be reliable. The authors recommend that, in these patients, the evaluation of glaucomatous damage be based on the GCIPL thickness map analysis rather than on the pRNFL thickness.
